# Transcription Factor KLF2 in Dendritic Cells Downregulates Th2 Programming via the HIF-1α/Jagged2/Notch Axis

**DOI:** 10.1128/mBio.00436-16

**Published:** 2016-06-14

**Authors:** Ye Xiong, Jerry B. Lingrel, Marcel Wüthrich, Bruce S. Klein, Neelakantan T. Vasudevan, Mukesh K. Jain, Mariam George, George S. Deepe

**Affiliations:** a Program in Pathobiology and Molecular Medicine, University of Cincinnati, Cincinnati, Ohio, USA; b Division of Infectious Diseases, College of Medicine, University of Cincinnati, Cincinnati, Ohio, USA; c Department of Molecular Genetics, Biochemistry & Microbiology, University of Cincinnati, Cincinnati, Ohio, USA; d Departments of Pediatrics, Medicine, and Medical Microbiology and Immunology, University of Wisconsin—Madison, Madison, Wisconsin, USA; e Case Cardiovascular Research Institute, Case Western Reserve University School of Medicine, Cleveland, Ohio, USA; f Program of Immunobiology, Cincinnati Children’s Hospital Medical Center, Cincinnati, Ohio, USA; g Veterans Affairs Hospital, Cincinnati, Ohio, USA

## Abstract

The adaptive immune response is tightly regulated by complex signals in dendritic cells (DCs). Although Th2 polarization is dictated by defined functional DC subsets, the molecular factors that govern the amplitude of these responses are not well understood. Krüppel-like factor 2 (KLF2) is a transcription factor that negatively regulates the activation of numerous immune cells in response to stimuli. Here, we demonstrate that suppression of KLF2 in conditioned DCs preferentially amplifies Th2 responses in two model systems, one of which is a prototypical intracellular pathogen and the other an allergen. This elevation in Th2 responses was dependent on contact-mediated Notch signaling *in vitro* and *in vivo*. A deficiency of KLF2 increased the expression of Notch ligand Jagged2 via hypoxia-inducible factor 1α (HIF-1α), which led to Th2 amplification. Our results revealed a novel circuit in DCs for Th2 polarization that is governed by KLF2.

## IMPORTANCE

Dendritic cells are the key element that bridges innate and adaptive immunity. A complex and not-well-understood area in dendritic cell biology is the regulatory network that predetermines or moderates their function to shape the adaptive immune response. Our study for the first time demonstrates that KLF2, a transcription factor, conditions dendritic cells to regulate Th2 responses via a Jagged2/Notch axis. Downregulation of KLF2 expression in dendritic cells may provide a beneficial effect for treatment of diseases such as obesity or parasitic infections but may be deleterious in the case of invasion by intracellular pathogens. Strategies to tune KLF2 may be useful for future therapeutic approaches to particular diseases of mankind.

## INTRODUCTION

Dendritic cells (DCs) are the most potent antigen-presenting cells. They connect innate and adaptive immunity to initiate, stimulate, and govern T cell responses in infections and immune disorders. Immature DCs patrol tissues and are activated upon sampling foreign antigens. After acquiring a mature phenotype, they migrate to lymph nodes (LNs), where they instruct T helper (Th) cell differentiation. Among the many Th cell subsets, Th1 cells are critical for host defense against intracellular pathogens, while Th2 cells are activated upon allergen exposure or parasitic infection (1).

The development of Th2 cells requires cooperative signals from DCs. Not only are cytokines like interleukin 4 (IL-4) determining factors for polarization, but molecules decorating DCs, such as the Notch ligand Jagged, influence Th2 activity (2). Although much is known about the signaling molecules of DCs that are engaged in Th2 differentiation, the regulators of these remain poorly characterized.

Krüppel-like factor 2 (KLF2) is a member of the Krüppel-like factor family of zinc finger transcription factors and is expressed in a variety of tissues and cells, including phagocytes and lymphocytes, to maintain quiescence (3). Downregulation of KLF2 unleashes a progression of inflammatory processes in several cell populations. In myeloid cells, bacterial cell wall components, hypoxia, and inflammatory cytokines reduce KLF2, licensing myeloid cell activation and aggravation of inflammatory responses (4).

Here, we report that KLF2 in DCs specializes in dictating Th2 programming in response to infection with a prototypical intracellular pathogen, *Histoplasma capsulatum*, and to an allergen, ovalbumin (OVA). A lack of KLF2 in DCs amplifies the production of IL-4 by Th2 cells. This effect was caused by increased numbers of Jagged2^+^ DCs. Inhibition of Notch signaling *in vitro* and *in vivo* eliminated the heightened production of IL-4. The induction of Jagged2 expression in DCs and Th2 is dependent on hypoxia-inducible factor 1α (HIF-1α). These findings reveal a previously unrecognized KLF2/HIF-1α/Jagged2/Notch axis that controls the magnitude of the Th2 response.

## RESULTS

### Myeloid KLF2 deficiency impedes fungal clearance via elevation of IL-4.

In murine histoplasmosis, a Th1-dominant immune response emerges between days 7 and 14 and is required to activate macrophages (Mϕs) for resolution. The products of Th2 override Th1 immunity, dampen Mϕ activation, and confer a permissive environment for intracellular fungal growth (5, 6). Mϕs devoid of KLF2 exhibit enhanced bactericidal activity, and a greater proportion of mice lacking this transcription factor in myeloid cells survive sepsis (4, 7). Hence, we queried whether myeloid KLF2 deficiency would exert a similar effect with an intracellular pathogen.

**FIG 1:**
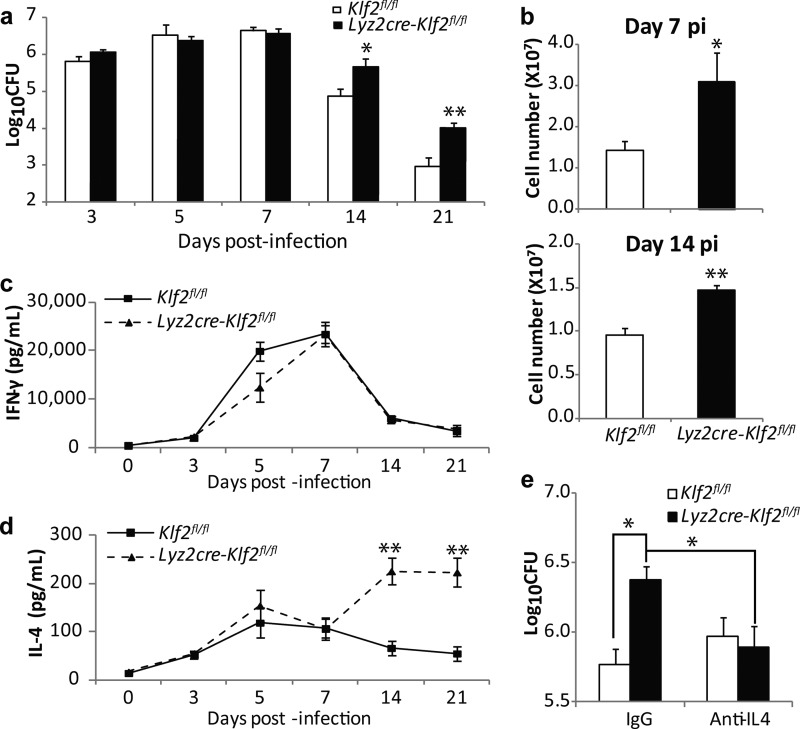
Fungal burdens and immune responses in *H. capsulatum*-infected mice. (a) Lung fungal burdens at days 3 to 21 p.i. in *Lyz2cre-Klf2*^fl/fl^ and control mice (*n* = 4 to 8). (b) Lung leukocytes at days 7 and 14 p.i. (*n* = 5 to 8). (c and d) IFN-γ (c) and IL-4 (d) concentrations in lung homogenates (*n* = 5 to 11, except *n* = 3 at day 5 p.i.). *, *P* < 0.05, and **, *P* < 0.01, for comparison with the results for control mice by *t* test. (e) Fungal burdens at day 14 p.i. in the lungs of mice treated with IgG or anti-IL-4 Ab (1 mg/mouse) (*n* = 6). *, *P* < 0.05, and **, *P* < 0.01, by two-way ANOVA. Data represent the mean results ± standard errors of the means (SEM).

We challenged *Lyz2cre-Klf2*^flox/flox^ mice (referred to as *Lyz2cre-Klf2*^fl/fl^ or KLF2^−/−^ mice) and *Klf2*^fl/fl^ (referred to as KLF2^+/+^) mice with a sublethal inoculum of *H. capsulatum* yeast cells and examined the fungal burdens in the lungs at serial intervals up to day 21 postinfection (p.i.). Similar numbers of yeast cells were recovered from mice in the two groups from days 3 to 7 p.i. The burdens in mutant mice were higher than those in infected controls (*P* < 0.01) at days 14 and 21 (Fig. 1a). Infection ultimately resolved in both groups.

We speculated that a diminished inflammatory cell response or an alteration in the cytokine environment accounted for the delayed resolution. The numbers of leukocytes were elevated in the lungs of *Lyz2cre-Klf2*^fl/fl^ mice at days 7 and 14 p.i. compared to the results for the controls; monocytes, Mφs, and neutrophils were the types primarily increased (Fig. 1b; see also Fig. S1a and b in the supplemental material). The magnified inflammation present in the lungs of *H. capsulatum*-infected *Lyz2cre-Klf2*^fl/fl^ mice correlated with a lower level of expression of *Klf2* and upregulation of chemoattractants (see Fig. S1c). We assessed multiple cytokines involved in host control of histoplasmosis, specifically those related to adaptive immunity. At day 14 p.i., the level of IL-4 was fourfold higher in the lungs of *Lyz2cre-Klf2*^fl/fl^ mice than in the lungs of the controls, whereas the levels of gamma interferon (IFN-γ), IL-17, IL-12, IL-6, and IL-1β were similar to the levels in the controls (Fig. 1c and d; see also Fig. S1c and d). We treated the animals with anti-IL-4 antibody (Ab) or IgG on day 7 p.i. and examined the fungal burdens in the lungs at day 14 p.i. Neutralizing IL-4 reduced the fungal burdens in the lungs of *Lyz2cre-Klf2*^fl/fl^ mice to the levels in the infected controls (Fig. 1e).

### KLF2-deficient myeloid cells amplify Th2 responses during *H. capsulatum* infection and OVA sensitization.

**FIG 2:**
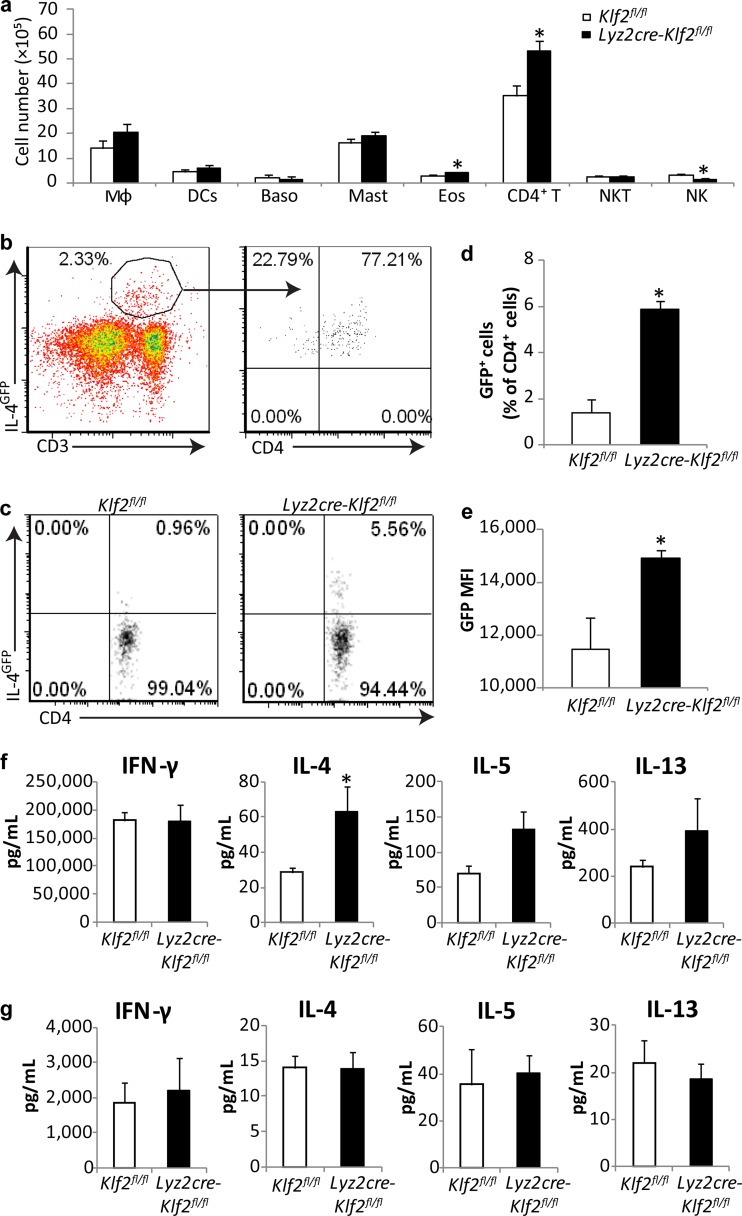
Myeloid KLF2 deficiency enhances Th2 responses in *H. capsulatum*-infected mice. (a) IL-4-producing cells in the lungs at day 14 p.i. were captured by IL-4 secretion assay (*n* = 4 or 5). Cell profiles are described in Materials and Methods. Baso, basophils; Mast, mast cells; Eos, eosinophils. (b to e) Percentages and MFI values of IL-4^GFP+^ cells in the lungs at day 14 p.i. (*n* = 5). (f and g) Th1/Th2 cytokine production by CD4^+^ cells (f) or CD4^−^ cells (g) isolated from the lungs at day 14 p.i. and restimulated for 24 h *ex vivo* (*n* = 4 or 5). *, *P* < 0.05, and **, *P* < 0.01, for comparison with the results for control mice by *t* test. Data represent the mean results ± SEM.

Elevated IL-4 coincided temporally with the emergence of adaptive immunity. We reasoned that Th2 cells were the principal source. Utilizing an IL-4 secretion assay, we captured CD4^+^ T cells as the main IL-4-producing population (Fig. 2a). The total number of IL-4^+^ CD4^+^ T cells was higher in *Lyz2cre-Klf2*^fl/fl^ mice than in controls. Although other populations, such as Μϕs and eosinophils, were captured, they were similar in numbers between the two groups.

We crossed *Lyz2cre-Klf2*^fl/fl^ mice to bicistronic IL-4^GFP^ reporter mice, which express green fluorescent protein (GFP)-labeled IL-4, to visualize IL-4 expression by CD4^+^ cells at day 14 p.i. This approach affirmed that CD4^+^ T cells were the dominant IL-4^+^ population (Fig. 2b). Infection of *Lyz2creKlf2*^fl/fl^ mice was associated with increased numbers of IL-4^GFP+^ CD4^+^ T cells in the lungs and an elevated IL-4^GFP^ mean fluorescence intensity (MFI) (Fig. 2c to e).

Since the engineered IL-4 gene allows the assessment of transcription, we sought to determine whether CD4^+^ T cells from *Lyz2cre-Klf2*^fl/fl^ mice produced greater quantities of IL-4. Leukocytes from the lungs and draining LNs of mice at day 14 p.i. were fractionated into CD4^+^ and CD4^−^ populations. Equal numbers from each group were stimulated *ex vivo* for 24 h with anti-CD3 and anti-CD28 Abs or phorbol myristate acetate (PMA) and ionomycin, respectively, and the supernatants were assayed for IFN-γ, IL-4, IL-5, and IL-13. In accordance with the elevated IL-4^GFP^ signal, lung CD4^+^ cells from *Lyz2cre-Klf2*^fl/fl^ mice produced more IL-4 than controls, but not more IFN-γ (Fig. 2f). The levels of IL-5 and -13 were increased, but not significantly. Lung CD4^−^ cells from both groups released low levels of Th1 or Th2 cytokines (Fig. 2g). Higher levels of IL-4, -5, and -13 (*P* < 0.01) were produced by the LN CD4^+^ cells from *Lyz2cre-Klf2*^fl/fl^ mice than by those from control mice (see Fig. S2a and b in the supplemental material).

In *Lyz2cre-Klf2*^fl/fl^ mice, the Th2 response was enhanced in a model that is heavily Th1 biased. To examine whether KLF2 downregulation exaggerated the Th2 response to an allergen, we utilized the acute OVA sensitization model. We examined the cells present in bronchoalveolar lavage (BAL) fluid samples from OVA-challenged mice. Myeloid KLF2 deficiency was associated with a higher number of inflammatory cells in the BAL fluid (see Fig. S3a and b in the supplemental material). IL-4 and -13 but not IFN-γ were elevated in the lungs of *Lyz2cre-Klf2*^fl/fl^ mice (see Fig. S3c). To determine whether the increased levels of IL-4 and -13 in OVA-sensitized *Lyz2cre-Klf2*^fl/fl^ mice were the consequence of a Th2 response, we isolated CD4^+^ cells from lungs and LNs and stimulated them with anti-CD3 and anti-CD28 Abs. The cells from *Lyz2cre-Klf2*^fl/fl^ mice produced more IL-4 and -13 than the cells from controls (see Fig. S3d).

### Loss or gain of KLF2 in DCs conditions Th2 polarization.

We hypothesized that KLF2 deficiency in DCs modifies signals that enhance Th2 responses. We first checked the lungs of infected *Lyz2cre-Klf2*^fl/fl^ and control mice for known stimulants of type 2 immunity, including IL-33, thymic stromal lymphopoietin (TSLP), and IL-25. None of these differed between the two groups (data not shown).

**FIG 3:**
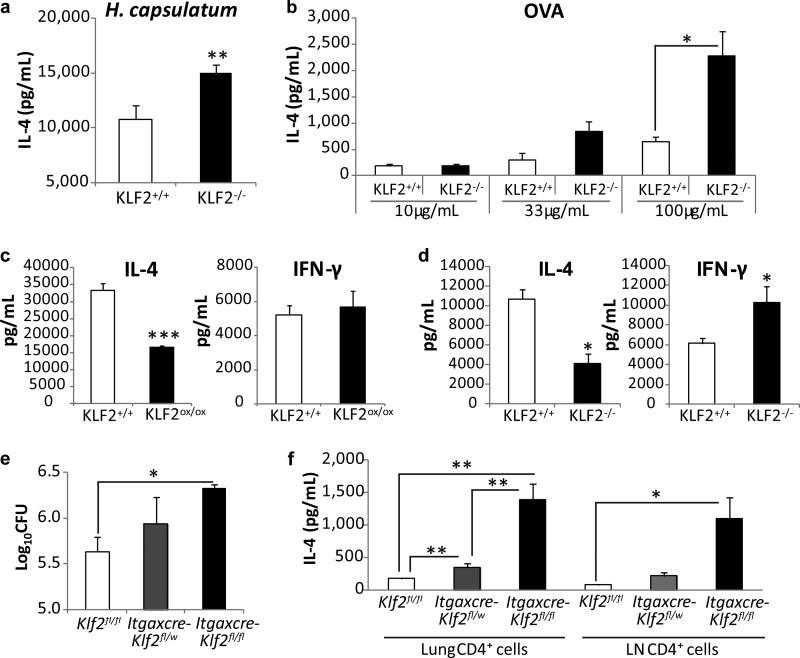
KLF2 in DCs dictates the strength of the Th2 response. (a and b) IL-4 production by naive cognate T cells after 5 days of coculture with 2 HK *H. capsulatum* yeast cells/BMDC (a) or 100-µg/ml OVA-pulsed BMDCs (b) and an additional 24 h of restimulation (summary of 4 to 6 experiments). *, *P* < 0.05, and **, *P* < 0.01, for comparison with the results for the control by Student’s *t* test. (c) IFN-γ and IL-4 production by T cells cocultured with KLF2^ox/ox^ BMDCs (3 experiments). (d) IL-4 and IFN-γ production by T cells cocultured with BMMφs (3 experiments). (e) Lung fungal burdens in control, *Itgaxcre-Klf2*^fl/w^ (w = wild-type allele), and *Itgaxcre-Klf2*^fl/fl^ mice at day14 p.i. (*n* = 4 or 5). (f) IL-4 production by CD4^+^ cells isolated from the lungs and LNs of *H. capsulatum*-infected mice at day 14 p.i. and restimulated for 24 h *ex vivo* (*n* = 5 or 6). *, *P* < 0.05, and **, *P* < 0.01, by Student’s *t* test or one-way ANOVA. Data represent the mean results ± SEM.

Among DC subsets, the CD11b^+^ population is key for shaping the Th2 phenotype (8). We used bone marrow-derived DCs (BMDCs) to investigate whether downregulation of KLF2 conditions DCs to promote Th2 activity. BMDCs obtained from *Klf2*^fl/fl^ (KLF2^+/+^) and *Lyz2cre-Klf2*^fl/fl^ (KLF2^−/−^) mice were cocultured with naive T cells from T cell receptor (TCR) transgenic 1807 (9) or OT-II mice, whose cells respond to *H. capsulatum* antigens or OVA, respectively. KLF2^−/−^ BMDCs incubated with heat-killed (HK) *H. capsulatum* or OVA greatly enhanced IL-4 release by cognate T cells (Fig. 3a and b). This increment was not a result of differences in T cell proliferation. T cells expanded similarly whether they were incubated with KLF2^+/+^ or KLF2^−/−^ DCs (see Fig. S4a and b in the supplemental material). Thus, KLF2 was instrumental in cytokine generation but not proliferation of T cells.

As a corollary, we explored the impact of KLF2 gain of function on the magnitude of the Th2 response. We utilized KLF2-overexpressing (KLF2^ox/ox^) BMDCs from KLF2 transgenic mice and cocultured them with naive T cells. The level of KLF2 mRNA was ~threefold higher in KLF2^ox/ox^ BMDCs than in KLF2^+/+^ DCs, and this increase was stable with or without stimuli (see Fig. S5 in the supplemental materials). BMDCs were pulsed with HK *H. capsulatum* for 24 h, followed by coculture with naive T cells from 1807 mice. While the level of IFN-γ production by T cells was comparable, the level of IL-4 secretion was lower in T cells incubated with KLF2^ox/ox^ DCs (Fig. 3c).

Apart from myeloid DCs, *Lyz2cre* drives the deletion of KLF2 in Mϕs that can present antigen to T cells. We cocultured KLF2^−/−^ bone marrow-derived Mϕs (BMMϕs) with T cells to determine whether these phagocytes promoted Th2 responses. T cells incubated with KLF2^−/−^ Μϕs generated less IL-4 but more IFN-γ than their KLF2^+/+^ counterparts (Fig. 3d).

To validate that DCs were responsible for the Th2 bias *in vivo*, we crossed *Itgaxcre* (*CD11ccre*) mice with *Klf2*^fl/fl^ mice. *Itgaxcre-Klf2*^fl/fl^ mice challenged with *H. capsulatum* displayed higher lung fungal burdens and more IL-4 than infected controls at day 14 p.i. (Fig. 3e). The levels of IL-4 production by sorted CD4^+^ cells from both the lungs and LNs of *Itgaxcre-Klf2*^fl/fl^ mice were considerably higher than those of controls (Fig. 3f).

### Myeloid KLF2 negatively regulates the strength of Th2 polarization via the Jagged2/Notch axis.

We performed a transwell assay to screen for the contribution of soluble signals to KLF2-mediated Th2 amplification. Naive T cells were cocultured with KLF2-sufficient or -deficient DCs separated by transwell for 5 days. T cells were harvested from each compartment and restimulated to measure the cytokines secreted (see Fig. S6a in the supplemental material). The level of IL-4 produced by T cells cocultured with KLF2^−/−^ DCs was higher (*P* < 0.05) than that of T cells cocultured with KLF2^+/+^ DCs, indicating that contact was required (Fig. S6b).

**FIG 4:**
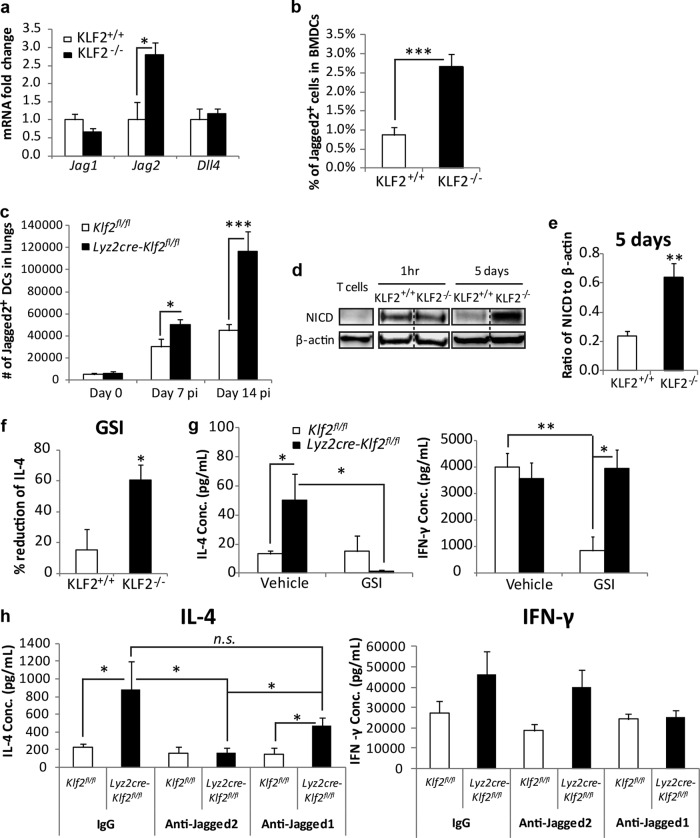
KLF2 deficiency enhances Th2 responses via the Jagged2/Notch axis. (a) Expression of Notch ligands in unstimulated BMDCs (*n* = 4 or 5). (b) Jagged2^+^ cell percentages in resting BMDCs (4 to 6 experiments). (c) Numbers of Jagged2^+^ DCs in the lungs of uninfected and *H. capsulatum*-infected mice (*n* = 5 to 9). (d and e) Western blot results (d) and densitometry analysis (e) of NICD in T cells after 1 h or 5 days of coculture with HK-*H. capsulatum*-loaded DCs (2 experiments). The dashed lines indicate that the two representative lanes were spliced together, although they were run on the same blot. (f) Percent changes in IL-4 levels with 50 µM GSI (DAPT) treatment during DC and T cell coculture (3 to 6 experiments). (g) IL-4 and IFN-γ in lung homogenates harvested at day 14 p.i. from *H. capsulatum*-infected mice treated with GSI (LY411,575) (*n* = 4 to 6). (h) Production of IL-4 and IFN-γ by activated lung CD4^+^ cells (CD3^+^ CD4^+^ CD69^+^) harvested at day 14 p.i. from the lungs of infected mice treated with anti-Jagged2 or anti-Jagged1 Ab. Cells were restimulated for 24 h *ex vivo* (*n* = 4 or 5). *, *P* < 0.05, **, *P* < 0.01, and ***P < 0.001, by Student’s *t* test or two-way ANOVA. Data represent the mean results ± SEM.

Membrane proteins on DCs are involved in priming an optimal Th2 response (10). Notch signaling is an important contact-dependent mechanism that regulates the differentiation of Th cells, including Th2 (11, 12). The DNA-binding protein recombining binding protein of hairless (RBP-J) is the principal mediator of Notch signaling and stimulates the expression of Th2 master regulator *Gata3* and *IL-4* (13, 14). Among the five canonical Notch ligands (Jagged1 and -2 and Delta-like ligand 1 [DLL1], DLL3, and DLL4) in mammals, DC Jagged1 or Jagged2 affects Th2 activity (12). KLF2 upregulates *Jag1* and represses *Jag2* in endothelial cells (15). We postulated that downregulation of KLF2 could increase Jagged2 to promote Th2 responses. In resting BMDCs, *Jag2* was upregulated in the absence of KLF2; *Jag1* and *Dll4* were unaltered (Fig. 4a). This increase correlated with a higher percentage of Jagged2^+^ cells in KLF2^−/−^ DCs, whereas the MFI was similar (Fig. 4b; Fig. S6c in the supplemental material). The differences in Jagged2^+^ cell percentages were maintained with OVA or *H. capsulatum* stimulation (Fig. S6d).

In the lungs of *Lyz2cre-Klf2*^fl/fl^ mice, the increase in Jagged2^+^ DCs appeared during infection. The numbers of Jagged2^+^ DCs were significantly elevated in *Lyz2cre-Klf2*^fl/fl^ mice at day 7 p.i. (*P* < 0.05), and the difference enlarged by day 14 p.i. (Fig. 4c). The proportions of two other Notch ligand-bearing DCs, DLL4^+^ and Jagged1^+^ DCs, were similar between groups at day 14 p.i. (see Fig. S6e and f in the supplemental material).

The activation of Notch licenses the tandem cleavage of Notch receptor by metalloprotease and γ-secretase to release its intracellular domain (NICD) that is required for its cellular function (16, 17). We postulated that the higher abundance of Jagged2^+^ cells among KLF2^−/−^ BMDCs would increase the proportion of responder cells to turn on Notch signaling and promote a Th2 response. To confirm Notch activation, we measured the amount of NICD in T cells after exposure to antigen-bearing BMDCs. In T cells cocultured with *H. capsulatum*-loaded KLF2-sufficient or -deficient BMDCs, NICD was induced as early as 1 h; however, this signal persisted for 5 days only in T cells cocultured with KLF2^−/−^ DCs (Fig. 4d and e). Correspondingly, *Hes1*, a direct target of canonical Notch signaling, was upregulated. High *Hes1* expression was maintained in T cells cocultured with KLF2^−/−^ DCs sharing the same well with T cells cocultured with KLF2^+/+^ DCs (see Fig. S6g in the supplemental material).

To examine the impact of Notch signaling on Th2 responses, we treated cells with a γ-secretase inhibitor (GSI). T cells cocultured with KLF2^−/−^ DCs loaded with HK *H. capsulatum* in the presence of GSI produced much less IL-4 than vehicle-treated cells (Fig. 4f). Only a slight reduction was observed in T cells cocultured with KLF2^+/+^ DCs. This phenotype was recapitulated with GSI treatment of mice. At day 14 of *H. capsulatum* infection, the elevated IL-4 in the lungs of *Lyz2Cre-Klf2*^fl/fl^ mice was abolished by GSI, while the level of IFN-γ was unchanged (Fig. 4g). Given the broad targets of GSI and to define the role of Jagged2 in governing the vigor of Th2 responses (18), we executed an *in vivo* experiment using anti-Jagged2 or anti-Jagged1 Ab. We sorted activated CD4^+^ T cells for *ex vivo* restimulation to examine the effect of anti-Jagged2 or anti-Jagged1 Ab treatment. As shown by the results in Fig. 4h, the administration of anti-Jagged2 Ab strikingly decreased the production of IL-4 by activated CD4^+^ T cells in *Lyz2Cre-Klf2*^fl/fl^ mice, without influencing IFN-γ. In contrast, anti-Jagged1 Ab treatment had an insignificant effect on the elevated Th2 response observed in KLF2-deficient mice.

**FIG 5:**
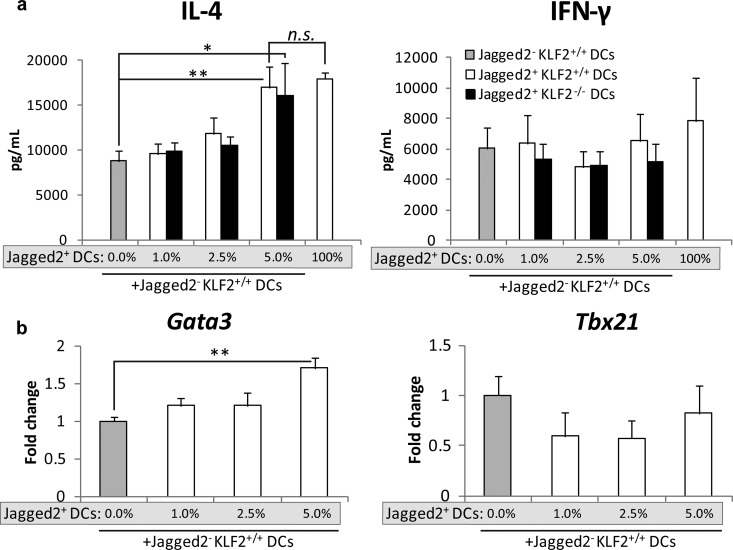
Jagged2-expressing DCs promote Th2 responses. (a) IL-4 and IFN-γ production by restimulated T cells after coculture with increasing percentages of Jagged2^+^ cells from KLF2^+/+^ or KLF2^−/−^ BMDCs loaded with HK *H. capsulatum* (4 experiments). (b) Th1/Th2 master regulator expression in cocultured T cells (3 experiments). *, *P* < 0.05, and **, *P* < 0.05, for comparison with the results for the control by one-way ANOVA. Data represent the mean results ± SEM.

To affirm that Th2 responses are tightly regulated by Jagged2-expressing DCs, we sorted BMDCs from *Lyz2cre-Klf2*^fl/fl^ and control mice into Jagged2^−^ and Jagged2^+^ populations. To wells containing Jagged2^−^ DCs (from KLF2^+/+^ mice), we added increasing percentages (1%, 2.5%, 5%, and 100%) of Jagged2^+^ DCs from either KLF2^+/+^ or KLF2^−/−^ mice to determine whether KLF2 deficiency in the Jagged2^+^ compartment had a functional advantage in Th2 polarization. These DCs were incubated with antigen and cocultured with naive T cells from 1807 transgenic mice. The addition of Jagged2^+^ DCs from KLF2^+/+^ mice increased IL-4 but not IFN-γ production by T cells in a dose-dependent manner (Fig. 5a). Maximal production of IL-4 was induced by 5% Jagged2^+^ DCs. *Gata3* was concomitantly induced with the addition of 5% Jagged2^+^ cells (Fig. 5b). The addition of Jagged2^+^ cells from either KLF2^+/+^ or KLF2^−/−^ mice generated a similar effect on cytokine production by T cells, suggesting that KLF2 limits the expansion of Jagged2^+^ DCs rather than endowing these cells with enhanced Th2-promoting activity.

### Th2 amplification by Jagged2-expressing DCs depends on HIF-1α.

**FIG 6:**
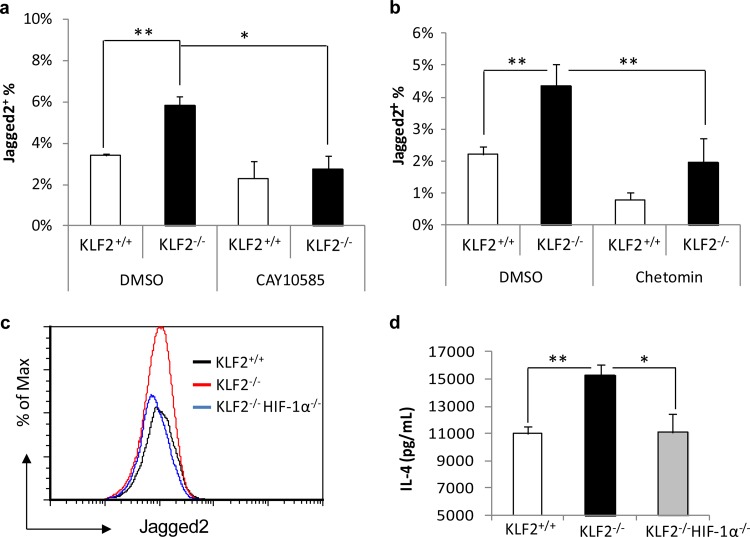
HIF-1α abrogated the induction of Jagged2^+^ population and Th2 by KLF2 deficiency. (a and b) Percentages of Jagged2^+^ cells in KLF2^+/+^ and KLF2^−/−^ DCs in the presence or absence of HIF-1α inhibitor (CAY10585, 30 µM) (a) or p300–HIF-1α complex inhibitor (chetomin, 7.5 nM) (b) during the last 3 days of BMDC differentiation (2 experiments). (c) Relative percentages of Jagged2^+^ cells in KLF2^+/+^, KLF2^−/−^, and KLF2^−/−^ HIF-1α^−/−^ BMDCs. (d) IL-4 production by restimulated T cells after incubation with Jagged2^−^ fraction of KLF2^+/+^, KLF2^−/−^, or KLF2^−/−^ HIF-1α^−/−^ BMDCs (with or without the addition of 5% Jagged2^+^ fraction) (2 experiments). *, *P* < 0.05, and **, *P* < 0.01, by two-way ANOVA. Data represent the mean results ± SEM.

KLF2 is known to negatively regulate HIF-1α in Mφs by preventing the formation of a complex with p300 (4). We found a higher level of HIF-1α in resting KLF2^−/−^ DCs (see Fig. S7a and b in the supplemental material). In parallel, more p300 accumulated in these cells (see Fig. S7c and d). Since HIF-1α directly regulates the expression of Jagged2 (19, 20), we speculated that KLF2 deficiency resulted in an unfettered HIF-1α function that subsequently increased Jagged2. We treated bone marrow cells with an inhibitor that prevents HIF-1α accumulation or p300–HIF-1α complex formation for 2 days prior to harvesting DCs. The percentage of Jagged2^+^ DCs was decreased by both means of HIF-1α blockade (Fig. 6a and b). Additionally, we crossed *Lyz2cre-Klf2*^fl/fl^ and *Lyz2cre-Hif1a*^fl/fl^ mice to create compound mutants. HIF-1α and KLF2 double deletion reduced the percentage of Jagged2^+^ DCs to the wild-type level (Fig. 6c). BMDCs from the compound mutants diminished the generation of IL-4 by T cells, but not their proliferation (Fig. 6d; see also Fig. S7e).

## DISCUSSION

Previous studies utilizing *Lyz2cre-Klf2*^fl/fl^ mice analyzed the function of KLF2 in combating bacterial infections by innate cells (4, 7). Downregulation of KLF2 upon stimulation with extracellular bacteria or bacterial products removes a repressive signal for the activation of Μϕs and release of proinflammatory cytokines. We reasoned that myeloid KLF2 deficiency might accelerate the resolution of *H. capsulatum* infection by promoting innate immunity. *Lyz2cre-Klf2*^fl/fl^ mice exhibited a nonlethal increased recruitment of inflammatory cells to the lungs. This phenotype is consistent with recent reports that microRNA or hypoxia signaling in KLF2-deficient Μϕs promotes inflammation (4, 21). However, fungal clearance in these mice was impeded, indicating no benefit of enhanced myeloid activation. Exacerbation of infection was attributable to enhanced production of IL-4 by CD4^+^ T cells and more Th2 in the lungs. KLF2 deficiency in myeloid cells created two alterations in the adaptive immune response that accounted for the amplification of nonprotective IL-4 generation.

The central observation that directed us to examine KLF2 in DCs was the finding that an increased fungal burden and elevated IL-4 developed coincidentally with adaptive immunity (22). Several lines of evidence implicated a deficiency of KLF2 in DCs as the primary defect for the increase in IL-4. First, using *Lyz2cre-Klf2*^fl/fl^ mice engineered with the IL-4^GFP^ reporter, Th2 cells were found to be the dominant producer, suggesting that an antigen-presenting cell was involved. Second, mice deficient in KLF2 in CD11c^+^ cells recapitulated the phenotype observed in the *Lyz2cre-Klf2*^fl/fl^ mice. Although KLF2 is absent in both DCs and alveolar Μϕs in the *Itgaxcre-Klf2*^fl/fl^ mice, the latter population is only involved in the innate response to *H. capsulatum* (23) and is unlikely to shape Th2 activity. Third, KLF2-deficient BMDCs stimulated T cells to produce more IL-4. This result supports a direct action of KLF2-deficient DCs in enhancing Th2 activity.

DCs are heterogeneous populations that contain functionally different and/or redundant subsets. The development of a Th2 response in parasitic infections, allergic airway inflammation, or atopic dermatitis is frequently associated with CD11b^+^ conventional DCs (cDCs) and monocyte-derived DCs (moDCs) (24). These two DC subsets synergize for optimal Th2 responses (8). The removal of *Klf2* by *Itgaxcre* in mice targets both cDCs and moDCs, while *Lyz2Cre* deletes genes only in moDCs. The amount of IL-4 produced by CD4^+^ cells from *Itgaxcre-Klf2*^fl/fl^ mice exceeded that generated by this population from *Lyz2cre-Klf2*^fl/fl^ mice. This finding suggests that elimination of KLF2 in cDCs and moDCs produces a stronger Th2 bias. The strength of the Th2 response was dependent on the number of *Klf2* alleles deleted in DCs. Thus, KLF2 in DCs acted as a rheostat to dictate the magnitude of Th2 responses.

In infected *Lyz2cre-Klf2*^fl/fl^ *Il4^Gfp^* mice, Th2 accumulated in the lungs and IL-4 production by individual cells was augmented. KLF2 regulates the production of several chemokines in T cells, and it is possible that it has a similar effect in DCs. Thus, enhanced recruitment of Th2 is likely a consequence of the augmented generation of Th2 chemoattractants by KLF2-deficient moDCs (8, 25). The production of these molecules may be caused by enhanced production by individual cells, by elevated numbers of moDCs in the lungs of *Lyz2cre-Klf2*^fl/fl^ mice, or by both. However, the production of chemoattractants is selective, since we did not see an elevation in Th1 cells releasing more IFN-γ. Another explanation for the accumulation of Th2 cells is prolonged survival and/or expansion. Notch signaling is a prosurvival factor for CD4^+^ T cells (26). Thus, Notch activation may operate on several levels to enhance Th2 cytokines, including direct amplification, enhanced recruitment, and prolonged survival.

One possible explanation for the elevations in Th2 cell numbers and activity in the *Lyz2cre-Klf2*^fl/fl^ mice is that type 2 innate lymphoid cells (ILC2s) drove the emergence of the type 2 immune cells (27). While we cannot exclude a role for ILC2s, we consider this scenario unlikely, since we did not observe any elevations in the levels of IL-5, IL-13, IL-25, IL-33, or TSLP in the lungs. Moreover, we would have expected the Th2 cells to emerge earlier if ILC2s were involved, because their activation is often early after antigen exposure.

The inflation of IL-4 by KLF2^−/−^ DCs was irrespective of the nature of the antigen, since OVA and *H. capsulatum* produced similar responses. To determine how KLF2 in DCs influenced Th2 activity, we tested the cytokines and cell surface molecules involved in this action. The essential cytokine for polarizing Th2, IL-4, is not produced by DCs, although KLF2 regulates it in T cells (28). Accessory molecules, including CD80/86, ICOSL, OX40L, and Jagged, reinforce Th2 differentiation (12, 29–31). With the exception of Jagged, they boost the proliferation of T cells (32). The presence or absence of KLF2 in DCs did not affect T cell proliferation. Thus, the vigor of proliferation and IL-4 generation were uncoupled. Since the levels of proliferation were similar between groups, we considered Jagged to be a likely candidate to explain enhanced IL-4.

Jagged proteins interact with Notch receptors on T cells to activate downstream signaling; this axis stimulates *de novo* synthesis of IL-4 by naive T cells and is sustained via a positive feedback loop (12, 13, 33). We demonstrated that more Jagged2^+^ DCs were found in uninfected BMDCs and the lungs of *Lyz2cre-Klf2*^fl/fl^ mice upon *H. capsulatum* infection. The addition of only 5% Jagged2^+^ DCs to Jagged2^−^ cells augmented IL-4 production by CD4^+^ T cells. Our findings indicate that KLF2 inhibited the expression of Jagged2 in DCs and that when KLF2 was absent, that inhibition was removed. This alteration leads to an expansion of Jagged2-bearing DCs.

The induction of Jagged2 on DCs in the absence of KLF2 was dependent on the activity of HIF-1α. Chemical inhibition of HIF-1α signaling or gene deletion reduced the proportion of Jagged2-bearing DCs. This finding is in line with studies showing that HIF-1α regulates Jagged2 expression (19). The net effect is a reduction in the percentage of Jagged2^+^ DCs and compromised Th2 amplification. Therefore, this transcription factor serves as an intermediate of the KLF2/Jagged2 axis for Th2 enhancement. This finding is congruent with a previous observation that hypoxia-conditioned DCs polarize Th2 cells (34).

While Notch signaling is indispensable for mounting an optimal Th2 response (11, 35), others have reported that Jagged2 in DCs is unnecessary for differentiation of Th2 cells or for the magnitude of IL-4 production (32, 36). The latter result differs from our own findings. A few considerations may explain the differences between our study and others. First, Notch activation is regulated by the availability of ligand or receptor (37), and Notch activation in CD4^+^ T cells can be influenced by a small percentage of Notch ligand-expressing DCs (38). One study employed overexpression of Jagged2 (~1,000-fold the level in the control) to explore its impact on Th2 responses. In this scenario, the availability of Notch receptors on T cells becomes a limiting factor in the activation of this pathway. Internalization of the ligand-receptor complex is requisite for Notch signaling. An overabundance of unoccupied Notch ligand retards endocytosis/recycling and dampens the magnitude of Notch activation (39, 40). The possibility that Notch signaling is not enhanced when ligand is in excess may be considered a reason for the unaltered Th2 responses. In our study, the amounts of Jagged2 decorating the cell surface did not differ between the two groups of DCs, but the proportion of Jagged2^+^ DCs was higher with KLF2 deficiency. KLF2^−/−^ DCs produced a sustained, not transient, Notch activation, as evidenced by positive staining of NICD and *Hes1* upregulation at the end of 5 days of coculture. This finding suggests that a continuous endocytosis/recycling of the ligand-receptor complex might be required for supporting Th2 amplification. Therefore, the higher percentage of Jagged2^+^ cells among KLF2^−/−^ DCs may be more active in triggering and maintaining Notch activation.

In summary, we identified KLF2 as a key determinant of the behavior of DCs in their interaction with T cells, as illustrated in Fig. S8 in the supplemental material. Exploration of this pathway uncovered a new regulatory mechanism in which KLF2 negatively governs the expression of Jagged2 on DCs. The outcome of this KLF2/HIF-1α/Jagged2/Notch axis is an alteration of Th2 activity. While this result may be detrimental for defense against intracellular pathogens like *H. capsulatum*, it could be salutary for defense against parasitic diseases or prevention of obesity, in which type 2 immunity is paramount. Thus, therapeutics directed at tuning KLF2 expression in DCs might provide a means to sway the immune response to a Th1 or Th2 response.

## MATERIALS AND METHODS

### Mice.

C57BL/6 mice were purchased from Jackson Laboratories (Bar Harbor, ME). *Lyz2Cre-Klf2*^fl/fl^ mice were obtained from Jerry Lingrel. Littermate *Klf2*^fl/fl^ mice served as controls. *Lyz2Cre-Klf2*^fl/fl^ *Il4^Gfp^* mice were generated by crossing C57BL/6 IL-4^GFP^ reporter (4-get) mice with *Lyz2Cre-Klf2*^fl/fl^ mice. *Itgaxcre-Klf2*^fl/fl^ mice were obtained by crossing *Itgaxcre* (Jackson Laboratory) and *Klf2*^fl/fl^ mice. OT-II mice were provided by Edith Janssen (Cincinnati Children’s Hospital Medical Center). KLF2 transgenic mice were maintained in the animal facility at Case Western Reserve University by Mukesh Jain. Double knockout (*Lyz2Cre-Klf2*^fl/fl^ *HIF1a*^fl/fl^) mice were generated by in-house breeding with *Lyz2Cre-HIF1a*^fl/fl^ mice (a gift from Timothy Eubank). The animals were housed in isolator cages and were maintained by the Department of Laboratory Animal Medicine, University of Cincinnati, which is accredited by the Association for Assessment and Accreditation of Laboratory Animal Care. All animal experiments were performed in accordance with the Animal Welfare Act guidelines of the National Institutes of Health, and all protocols were approved by the Institutional Animal Care and Use Committee of the University of Cincinnati.

### *H. capsulatum* infection.

*H. capsulatum* yeast strain G217B was grown for 72 h at 37°C. Animals were inoculated with 2 × 10^6^ yeast cells in ~35 µl of HBSS. Organ culture of *H. capsulatum* and isolation of leukocytes were performed as described previously (41).

### IL-4 secretion assay.

An IL-4 secretion assay (Miltenyi Biotec, Auburn, CA) was performed following manufacturer's instruction. Briefly, leukocytes were harvested from day 14 *H. capsulatum*-infected lungs and restimulated with equal numbers of HK *H. capsulatum* yeast cells overnight. The cells were then processed according to the manufacturer’s protocol and analyzed by flow cytometry.

### Antibody neutralization and chemical inhibition.

For IL-4 neutralization, mice were given 1 mg of either rat IgG or anti-mouse IL-4 Ab (11B11) intraperitoneally (i.p.) at day 7 p.i. For Notch inhibition, GSI Ly411,575 (Sigma) or dimethyl sulfoxide (DMSO) was given i.p. at 7 mg/kg of body weight daily from day 7 p.i. In the Notch ligand blockade experiment, 0.25 mg of purified hamster IgG (Leinco Technologies, St. Louis, MO) or hamster anti-mouse Jagged1 or Jagged2 Ab (BioLegend, San Diego, CA) was given to mice i.p. at day 0 and at days 4 and 8 p.i. HIF-1α signaling was blocked with inhibitors against HIF-1α accumulation (30 µM CAY10585) (Cayman Chemical, Ann Arbor, MI) or p300–HIF-1α complex formation (7.5 nM chetomin) (Santa Cruz Biotechnologies, Dallas, TX**)** for 3 days prior to harvesting DCs.

### *Ex vivo* restimulation.

CD4^+^ cells from the lungs and LNs were sorted with magnetic beads (L3T4) (Miltenyi) and restimulated with 1-µg/ml plate-bound anti-CD3 Ab and 5-µg/ml soluble anti-CD28 Ab. The flowthrough (CD4^−^ cells) was restimulated with 50-ng/ml PMA and 500-ng/ml ionomycin. Amounts of 10^5^ CD4^+^ or CD4^−^ cells were replated in 150 µl medium, and supernatants were collected after 24 h. In the experiment with anti-Jagged2 Ab, activated CD4^+^ T cells (CD3^+^ CD4^+^ CD69^+^) harvested from LNs at day 14 p.i. were sorted using a FACSAria II (BD Biosciences, San Jose, CA). Isolated cells were stimulated for 24 h with anti-CD3 and anti-CD28 Abs *ex vivo*.

### Generation of bone marrow-derived DCs and Mϕs.

Bone marrow was isolated from the hind tibias and femurs of 8- to 10-week-old mice by flushing the bones with Hanks’ balanced salt solution (HBSS). Isolated cells were added at a concentration of 2 × 10^5^ cells/ml to 50 ml of RPMI 1640 supplemented with 10% fetal bovine serum, 0.1% gentamicin sulfate, and 0.005% 2-mercaptoethanol. Cells were treated with 10-ng/ml mouse granulocyte-Mϕ colony-stimulating factor (GM-CSF) (PeproTech, Rocky Hill, NJ) and cultured at 37°C in the presence of 5% CO_2_. The cells were supplemented with an additional 50 ml of medium and 10-ng/ml GM-CSF at days 3 and 6 after isolation. At day 6, the adherent cells (BMMϕs) were harvested. At days 8 to 10, the nonadherent cells were enriched for DCs using CD11c microbeads (Miltenyi Biotec). These cells were >90% CD11c^+^. In some experiments, BMDCs were stimulated with 100-µg/ml OVA or different multiplicities of *H. capsulatum* (0.5×, 1×, or 2× the number of DCs) for 24 h prior to assay.

### Coculture of BMDCs and naive T cells.

CD11c^+^ BMDCs were pulsed with 2 HK *H. capsulatum* yeast cells/DC or 100-ng/ml OVA for 24 h prior to coculture with naive T cells. Naive CD4^+^ T cells from 1807 Tg (*H. capsulatum* antigen TCR transgenic) mice (9) or OT-II mice were isolated with CD4 CD62L microbeads (Miltenyi Biotec). Antigen-bearing BMDCs (5 × 10^4^) were cocultured with naive T cells (1 × 10^5^) for 5 days. In experiments with an artificial mixture of Jagged2^−^ and Jagged2^+^ DCs, CD11c^+^ BMDCs were sorted by fluorescence-activated cell sorting (FACS) to collect Jagged2^−^ and Jagged2^+^ fractions. A concentration of 1%, 2.5%, or 5% Jagged2^+^ DCs was added to Jagged2^−^ DCs prior to the addition of HK *H. capsulatum*. At the end of 5 days, T cells (1 × 10^5^) were replated with anti-CD3 and anti-CD28 Abs for 24 h in 200 µl of medium. For the *in vitro* Notch blockade experiment, 50 µM of the GSI DAPT {*N*-[*N*-(3,5-difluorophenacetyl)-l-alanyl]-*S*-phenylglycine *t*-butyl ester} (Sigma) was added to the coculture medium.

### Other methods.

Other experimental procedures, including OVA sensitization, T cell proliferation, transwell study, flow cytometry, RNA isolation and cDNA synthesis, quantitative reverse transcription (qRT-PCR), assessment of cytokines/chemokines, and Western blotting, are described in detail in Text S1 in the supplemental material.

### Statistics.

Analysis of variance (ANOVA) was used to compare multiple groups with one or two variants, while the unpaired Student’s *t* test was used to compare two groups. A *P* value of less than 0.05 was considered statistically significant.

## SUPPLEMENTAL MATERIAL

Text S1  Supplemental materials and methods. Download Text S1, DOCX file, 0.02 MB

Figure S1  Inflammatory responses in the lungs of *H. capsulatum*-infected mice. (a and b) Leukocyte populations at day 7 (a) and day 14 (b) p.i. (*n* = 5 to 7). Cell profiles are described in “Flow cytometry” in Text S1 in the supplemental material. (c and d) Cytokine profiles at day 7 p.i.: expression levels in lung leukocytes normalized to day 0 leukocytes (c) and concentrations in lung homogenates (d) (*n* = 3 or 4). **, *P* < 0.05, and **, *P* < 0.01, for comparison with the results for control mice by Student’s *t* test. Data represent the mean results ± SEM. Download Figure S1, TIF file, 1.5 MB

Figure S2  Myeloid KLF2 deficiency enhances Th2 response in mice infected with *H. capsulatum*. Th1/Th2 cytokine production by CD4^+^ cells (a) or CD4^−^ cells (b) isolated from LNs harvested at day 14 p.i. and restimulated for 24 h *ex vivo* (*n* = 4 or 5). *, *P* < 0.05, and **, *P* < 0.01, for comparison with control mice by Student’s *t* test. Data represent the mean results ± SEM. Download Figure S2, TIF file, 0.7 MB

Figure S3  Myeloid KLF2 deficiency enhances Th2 responses in mice sensitized with OVA. (a and b) Leukocytes in BAL fluid 24 h after last OVA challenge (*n* = 5 to 7 in HBSS control, *n* = 11 to 13 in OVA-treated group). (c) Th1/Th2 cytokine levels in lung homogenates at day 18 after OVA sensitization (*n* = 5). (d) Production of IL-4 by CD4^+^ cells from lungs or LNs isolated at day 18 from OVA-sensitized mice and restimulated for 24 h *ex vivo* (*n* = 6). *, *P* < 0.05, and **, *P* < 0.01, for comparison with the results for control mice by Student’s *t* test. Data represent the mean results ± SEM. Download Figure S3, TIF file, 1.1 MB

Figure S4  T cell proliferation of naive T cells cocultured with antigen-loaded BMDCs. T cell proliferation of naive 1807 Tg mouse T cells cocultured with HK-*H. capsulatum*-loaded DCs (a) or OT-II T cells cocultured with OVA-pulsed DCs (b) at day 3 and day 5 of coculture (results shown are representative of 2 experiments). Download Figure S4, TIF file, 1.2 MB

Figure S5  KLF2 overexpression in BMDCs. KLF2 mRNA in KLF2^ox/ox^ BMDCs before and after 24 h of antigen stimulation (2 experiments). ***, *P* < 0.001, for comparison with the results for control DCs by Student’s *t* test. Data represent the mean results ± SEM. Download Figure S5, TIF file, 0.2 MB

Figure S6  KLF2 deficiency selectively enhances contact-dependent Th2 polarization via induction of Jagged2^+^ population. (a) Demonstration of transwell coculture system. (b) Production of Th1 and Th2 cytokines by T cells restimulated for 24 h after 5 days of coculture with either KLF2^+/+^ or KLF2^−/−^ OVA-pulsed BMDCs that were separated by 0.4-µm transwell membrane. Control (Ctrl) T cells were cocultured with KLF2^+/+^ (or KLF2^−/−^) BMDCs that were plated in both compartments of transwells (2 experiments). (c and d) Percentages of Jagged2^+^ BMDCs at rest (c) or after 24 h of exposure to 2 HK *H. capsulatum* yeast cells/DC or 100-µg/ml OVA (d) (3 experiments). (e and f) Proportions (e) and numbers (f) of Notch ligand^+^ DCs in the lungs of mice infected for 14 days (*n* = 3 or 4). (g) *Hes1* expression in T cells after 5 days of coculture with HK-*H. capsulatum*-loaded DCs in transwells (2 experiments). *, *P* < 0.05, and **, *P* < 0.01, for comparison with the results for the control by two-way ANOVA. Data represent the mean results ± SEM. Download Figure S6, TIF file, 2 MB

Figure S7  KLF2 regulates Th2 responses via HIF-1α signaling. (a and b) Western blot results (a) and densitometry analysis (b) of HIF-1α in BMDCs (3 to 4 experiments). (c and d) Confocal images (c) and fluorescence intensities (d) of p300 in BMDCs (2 experiments). (e) Fold proliferation of naive T cells after 5 days of coculture (2 experiments). **, *P* < 0.01 for comparison with the results for control DCs by Student’s *t* test. Data represent the mean results ± SEM. Download Figure S7, TIF file, 2.5 MB

Figure S8  Scheme of KLF2 signaling pathway in Th2 amplification. The absence of KLF2 increases the accumulation of HIF-1α and p300, promoting the expression of Jagged2. Surface Jagged2 on DCs can trans-interact with Notch receptors on T cells, mainly Notch1, thereby activating canonical Notch signaling via processes numbered as follows: (1) endocytosis of bound Jagged2 results in mechanical breakage of Notch receptor that exposes extracellular cleavage site on Notch receptor; (2 and 3) tandem cleavage by ADAM and γ-secretase; (4) release and nuclear translocation of NICD. In the nucleus, NICD binds to RBP-J and recruits coactivator and mastermind-like protein (MAML), hence promoting the expression of Gata3, IL-4, and Hes1. The production of IL-4 positively reinforces the Th2 polarization. Download Figure S8, TIF file, 0.9 MB
