# Supplementary material for: Transcription Factor KLF2 in Dendritic Cells Downregulates Th2 Programming via the HIF-1α/Jagged2/Notch Axis
Source: mBio. 2016 Jun 14;7(3):e00436-16. doi: 10.1128/mBio.00436-16 (PMC4916374; doi:10.1128/mBio.00436-16)
Supplement: Text S1 — Supplemental materials and methods. Download [file mbo003162840s1.docx]

**Supplemenatary Methods**

### OVA sensitization. Mice were sensitized on day 0, 7, and 14 with an intraperitoneal (i.p*.*) injection containing 100 μg of chicken egg OVA (Sigma, St Louis, MO, USA) suspended in 100 μL of Imject Alum (Thermo Scientific). All mice received 3 consecutive i.n. challenges with 50 μg of OVA in HBSS on days 15 to 17 and were sacrificed on day 18. HBSS with Alum was used as vehicle control.

**T cell proliferation**. Naïve T cells were labeled with 5μM CFSE at room temperature for 20min. At days 3 and 5 of co-culture with DCs, T cells were acquired and stained with CD4 antibody. CFSE fluorescence was then measured with flow cytometer. Cells were gated under CD4^+^ population for analysis of CFSE dilution.

**Transwell study**. Antigen bearing BMDCs were co-cultured with naïve T cells for 5 days. In the top chamber were the OVA pulsed KLF2^+/+^ BMDCs (1.25×10^5^) plus OT-II T cells (2.5×10^5^) and in the bottom chamber were the OVA pulsed KLF2^-/-^ BMDCs (3.75×10^4^) plus OT-II T cells (7.5×10^5^). These two chambers were separated by 0.4μm polyester transwell membrane. At the end of 5 days, T cells (1×10^5^) from each chamber were re-plated with anti-CD3 and anti-CD28 for 24 hr in 200μl of medium.

**Flow cytometry.** The phenotype of cells from mouse lungs was determined by incubating lung leukocytes with antibodies indicated below and CD16/32 to limit nonspecific binding. Leukocytes were stained at 4°C for 15 min in HBSS containing 1% bovine serum albumin (BSA). Cells were stained with combinations of the following antibodies: Ly6G, Ly6C, CD11b, F4/80, CD11c, I-A^b^, Siglec-F, CD3, CD4, CD8, NK1.1, FcεRI, Foxp3, CD49b, c-kit, Jagged1, Jagged2, DLL4 from BD Biosciences, ebioscience (San Diego, CA) or Biolegend (San Diego, CA). Cells were washed and resuspended in 1% paraformaldehyde. Experiments with appropriate isotype controls were performed in parallel. The analysis was performed utilizing an Accuri C6 (BD Biosciences) flow cytometer, and the results were analyzed with the FCS Express software. Cells were identified by following markers: Mφ (F4/80^+^, CD11b^+^), DCs (CD11c^+^, I-A^b+^), monocytes (Mono) (Ly6C^+^, CD11b^+^), PMN (Ly6G^+^, CD11b^+^), basophils (Baso) (CD49b^+^, FcεRI^+^), mast cells (c-kit^+^, FcεRI^+^), eosinophils (Eos) (CD11b^+^, Siglec-F^+^), NKT cells (NK1.1^+^, CD3^+^), NK cells (NK1.1^+^, CD3^-^), CD4+ T cells (CD3^+^, CD4^+^), CD8+ T cells (CD3^+^, CD8^+^), regulatory T cells (Tregs) (CD4^+^, Foxp3^+^).

**RNA isolation and cDNA synthesis.** Total RNA was isolated from whole lungs of mice using TRIzol (Invitrogen, Carlsbad, CA), or from cultured cells using RNeasy Mini Plus Kit (Qiagen, Limburg, Netherland). Oligo(dT)-primed cDNA was prepared by using the reverse transcriptase system (Promega) according to the manufacturer's instructions.

**Quantitative real-time PCR**. Quantitative real-time PCR for analysis of cytokine transcription was performed using TaqMan master mixture and primers obtained from Applied Biosystems (Foster City, CA). Samples were analyzed with an ABI Prism 7500 (Applied Biosystems). The hypoxanthine phosphoribosyl transferase 1 housekeeping gene was used as an internal control. The conditions used for amplification were 50°C for 2 min and 95°C for 10 min, followed by 40 cycles of 95°C for 15 s and 60°C for 1 min.

**Assessment of cytokines/chemokines.** Protein concentrations of IFN-γ and IL-4 were determined by ELISA (Thermo Scientific). Other cytokines/chemokines were measured by Milliplex assay using the MagPix (Millipore Corp., Billerica, MA).

**Western blot.** 5×10^5^ cells were lysed in 50μL cell denaturing buffer containing proteinase inhibitor (Thermo Scientific) on ice for 15min. Fifty μg of protein were electrophoresed on a 4%–20% Tris-glycine gradient gel (Thermo Scientific) and transferred onto nitrocellulose membrane (Millipore). The membrane was blocked with 5% non-fat milk in Tris-buffered saline containing 0.05% Tween 20 for 1 hr at room temperature. The membrane was incubated overnight at 4°C with antibody to Val1744 of Cleaved Notch1 (Cell Signaling) or HIF-1α (Novusbio). An HRP-conjugated goat anti-rabbit antibody was added. FluorChem HD2 imager was used to detect chemiluminescence after development with Luminata Forte western HRP substrate (Millipore). β-actin was a protein loading control.

**Microscopy.** Unstimulated DCs were placed on chamber slide (Millipore) for 24hr and fixed with 1% of paraformaldehyde for 30 min. Cells were sequentially incubated with p300 antibody (Santa Cruz Biotechnologies) overnight at 4°C, a Alexa 647-conjugated secondary antibody (life technologies, Carlsbad, [CA](https://www.google.com/search?safe=off&biw=1024&bih=677&q=Carlsbad+California&stick=H4sIAAAAAAAAAOPgE-LSz9U3MKmqSInPVeIAsYtMyvO0tLKTrfTzi9IT8zKrEksy8_NQOFYZqYkphaWJRSWpRcUA_pIQXEQAAAA&sa=X&ved=0ahUKEwiWor79_q7LAhXnr4MKHTJgDCkQmxMIhwEoATAO)) for 2hr at room temperature, then DAPI in mounting medium (Vector laboratories, Burlingame, CA) to adhere coverslip. Images were acquired on a Zeiss LSM710 confocal connected to Zeiss Axio-observer.Z1 inverted microscope and visualized using ZEN 2011 software.
