# Supplementary figures and images for: Transcription Factor KLF2 in Dendritic Cells Downregulates Th2 Programming via the HIF-1α/Jagged2/Notch Axis
Source: mBio. 2016 Jun 14;7(3):e00436-16. doi: 10.1128/mBio.00436-16 (PMC4916374; doi:10.1128/mBio.00436-16)

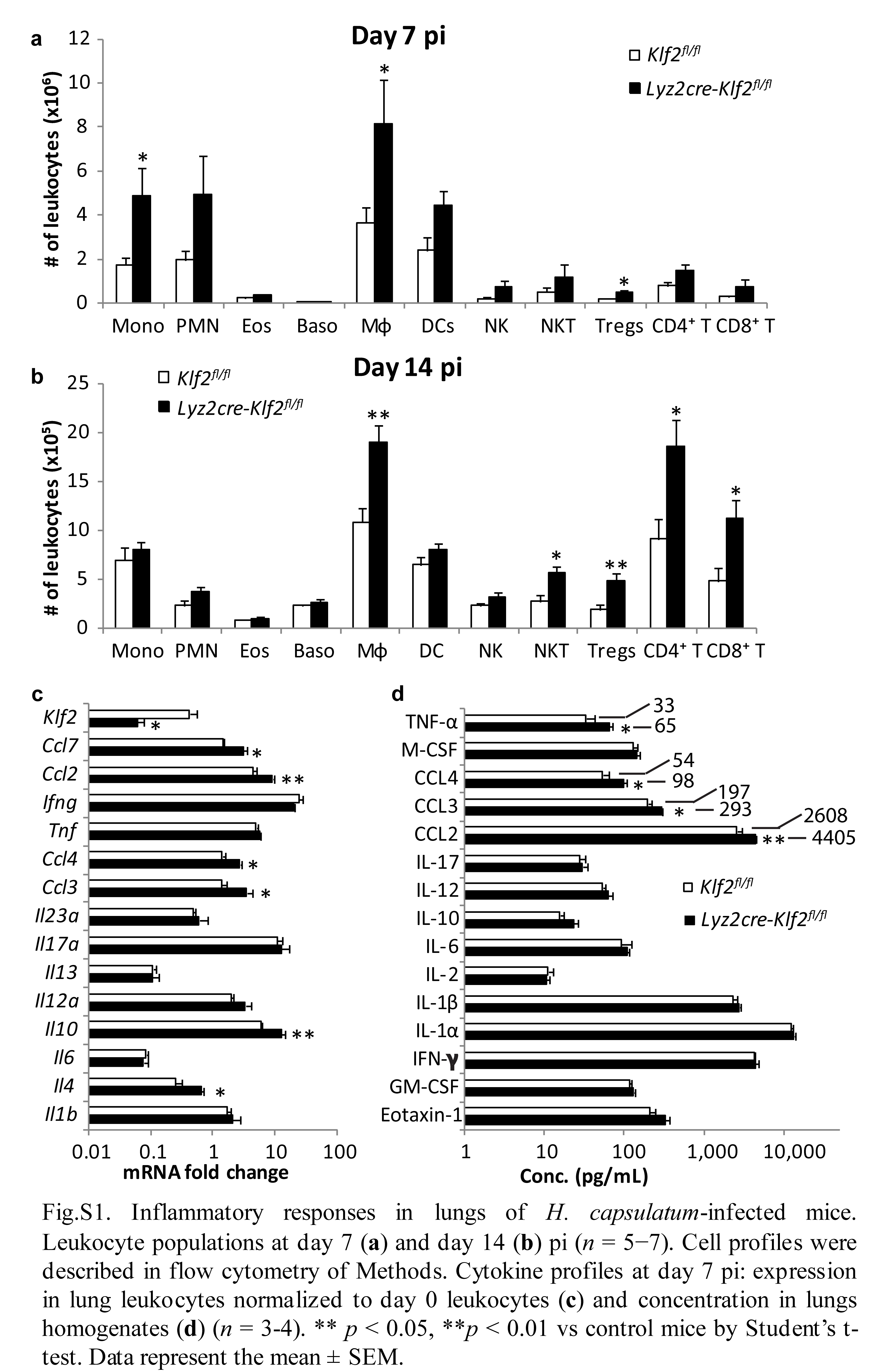

Supplement: Figure S1 — Inflammatory responses in the lungs of H. capsulatum-infected mice. (a and b) Leukocyte populations at day 7 (a) and day 14 (b) p.i. (n = 5 to 7). Cell profiles are described in “Flow cytometry” in Text S1 in the supplemental material. (c and d) Cytokine profiles at day 7 p.i.: expression levels in lung leukocytes normalized to day 0 leukocytes (c) and concentrations in lung homogenates (d) (n = 3 or 4). **, P < 0.05, and **, P < 0.01, for comparison with the results for control mice by Student’s t test. Data represent the mean results ± SEM. Download [file mbo003162840sf1.tif]

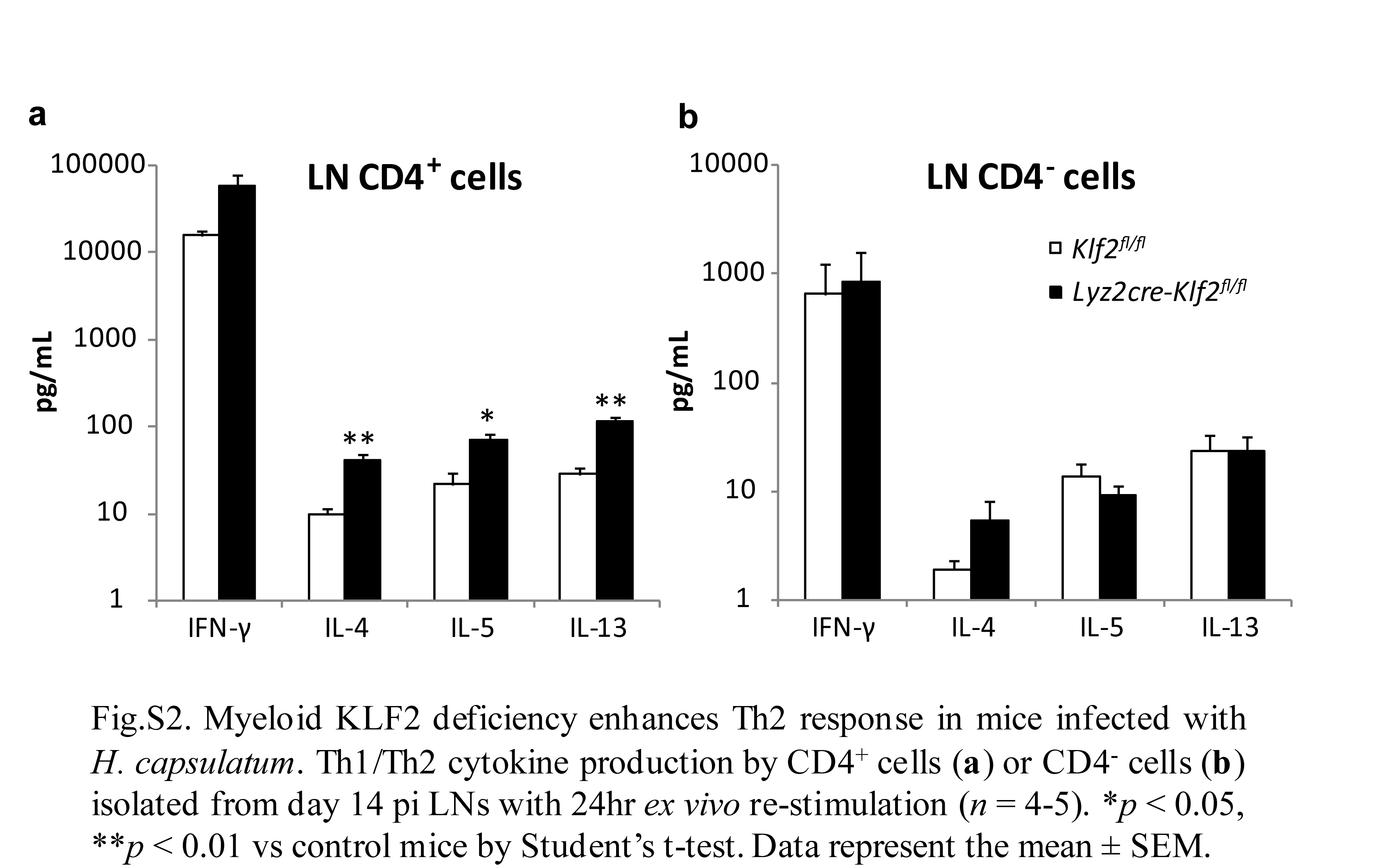

Supplement: Figure S2 — Myeloid KLF2 deficiency enhances Th2 response in mice infected with H. capsulatum. Th1/Th2 cytokine production by CD4+ cells (a) or CD4− cells (b) isolated from LNs harvested at day 14 p.i. and restimulated for 24 h ex vivo (n = 4 or 5). *, P < 0.05, and **, P < 0.01, for comparison with control mice by Student’s t test. Data represent the mean results ± SEM. Download [file mbo003162840sf2.tif]

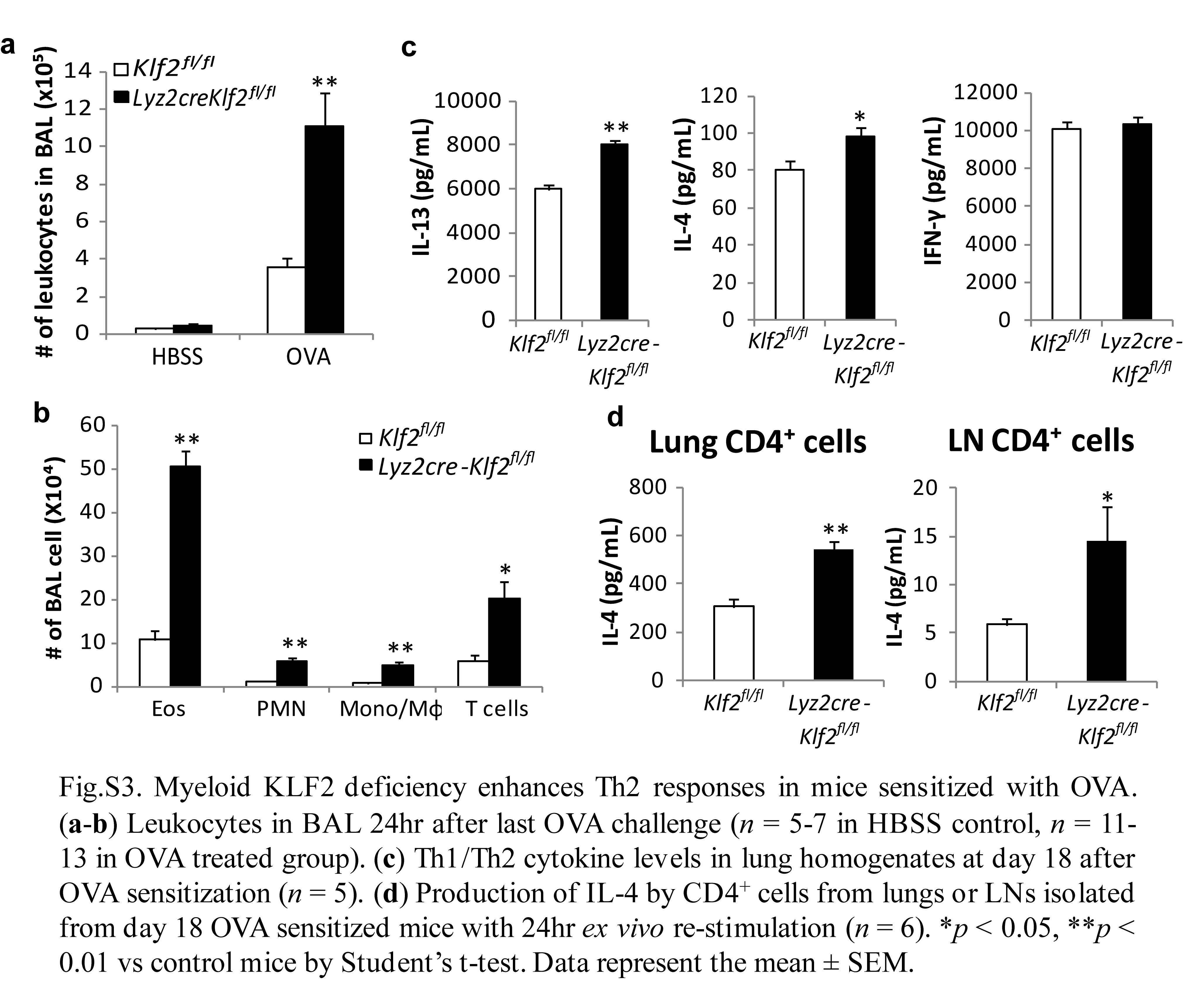

Supplement: Figure S3 — Myeloid KLF2 deficiency enhances Th2 responses in mice sensitized with OVA. (a and b) Leukocytes in BAL fluid 24 h after last OVA challenge (n = 5 to 7 in HBSS control, n = 11 to 13 in OVA-treated group). (c) Th1/Th2 cytokine levels in lung homogenates at day 18 after OVA sensitization (n = 5). (d) Production of IL-4 by CD4+ cells from lungs or LNs isolated at day 18 from OVA-sensitized mice and restimulated for 24 h ex vivo (n = 6). *, P < 0.05, and **, P < 0.01, for comparison with the results for control mice by Student’s t test. Data represent the mean results ± SEM. Download [file mbo003162840sf3.tif]

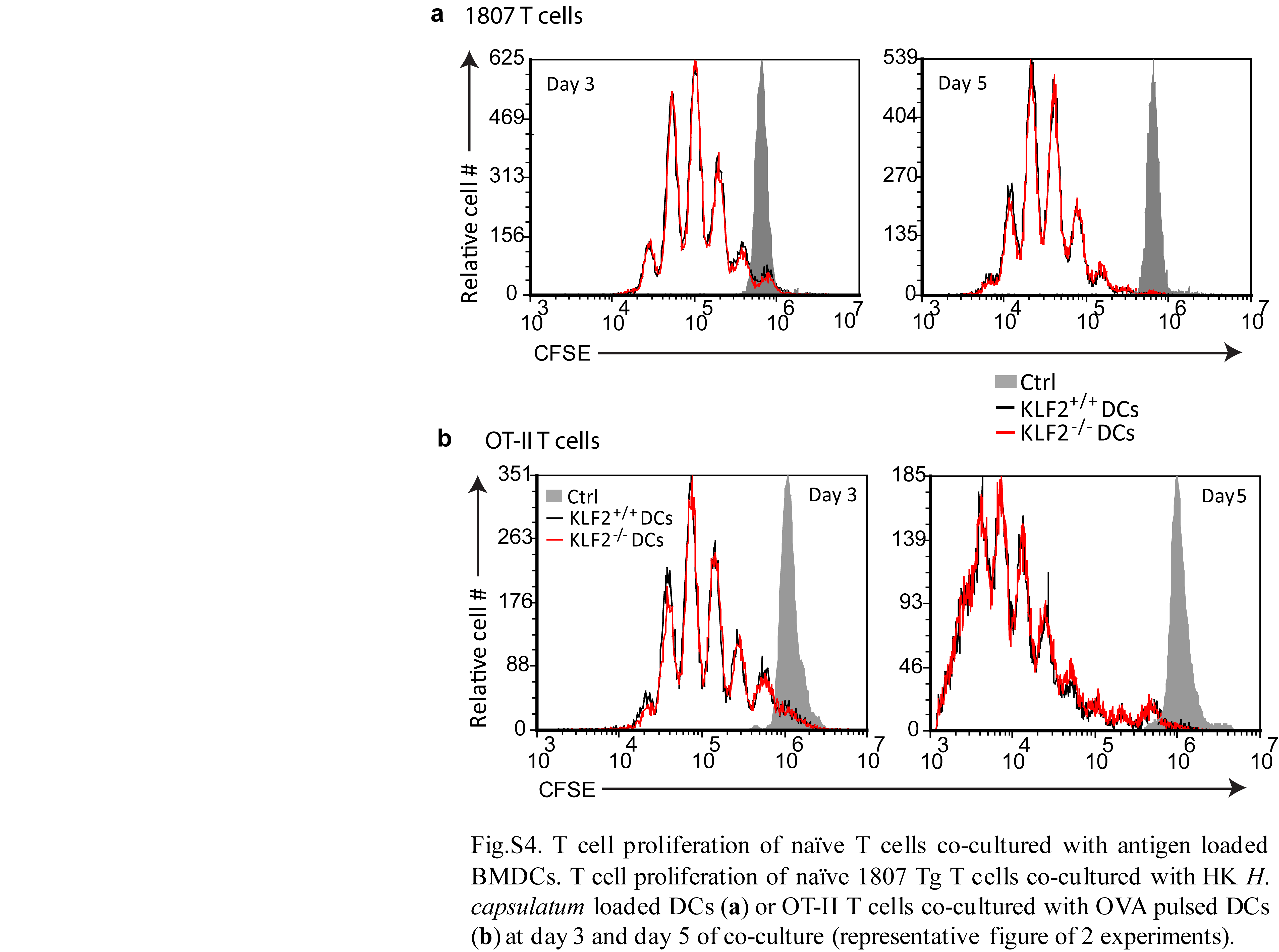

Supplement: Figure S4 — T cell proliferation of naive T cells cocultured with antigen-loaded BMDCs. T cell proliferation of naive 1807 Tg mouse T cells cocultured with HK-H. capsulatum-loaded DCs (a) or OT-II T cells cocultured with OVA-pulsed DCs (b) at day 3 and day 5 of coculture (results shown are representative of 2 experiments). Download [file mbo003162840sf4.tif]

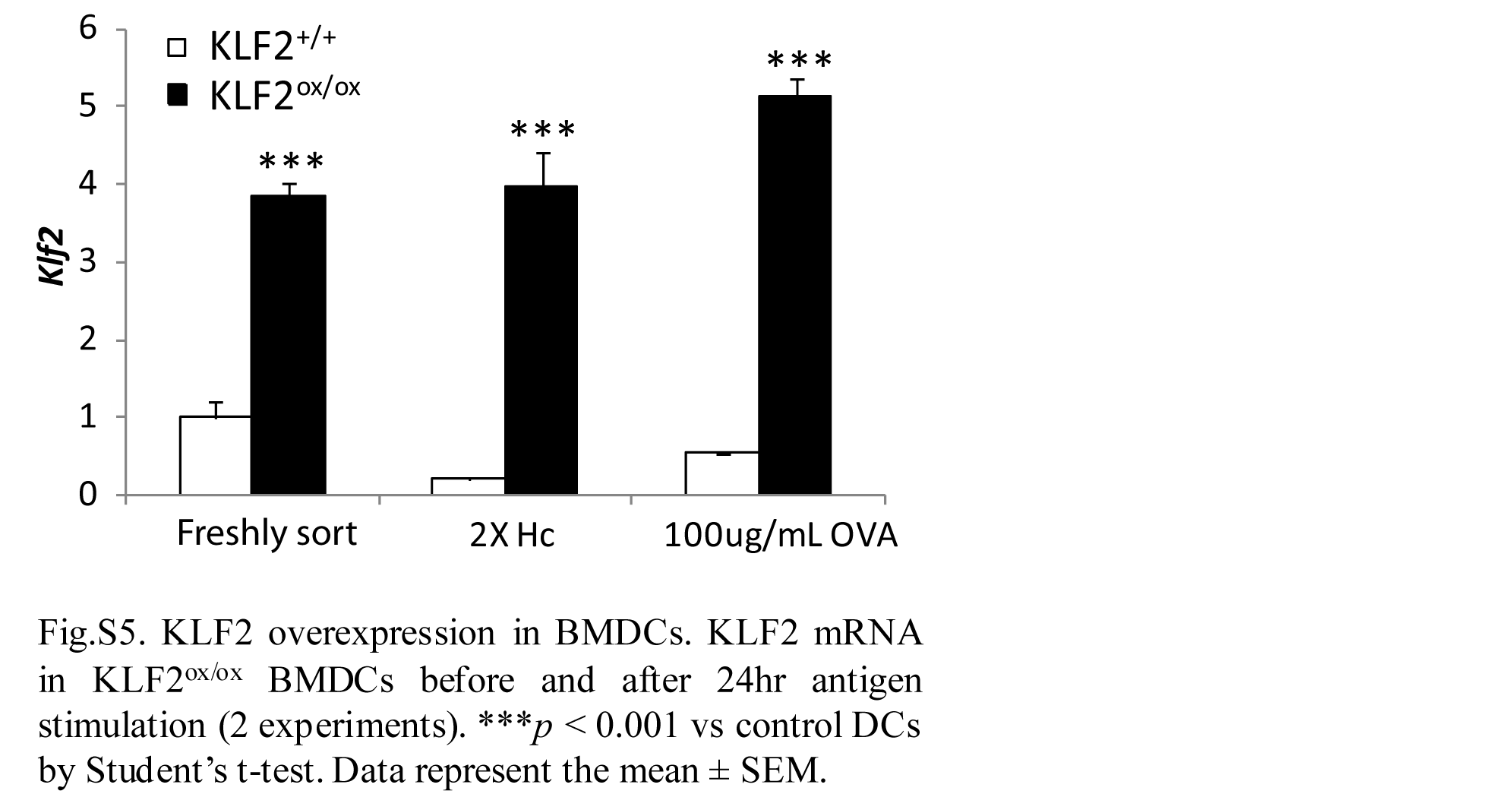

Supplement: Figure S5 — KLF2 overexpression in BMDCs. KLF2 mRNA in KLF2ox/ox BMDCs before and after 24 h of antigen stimulation (2 experiments). ***, P < 0.001, for comparison with the results for control DCs by Student’s t test. Data represent the mean results ± SEM. Download [file mbo003162840sf5.tif]

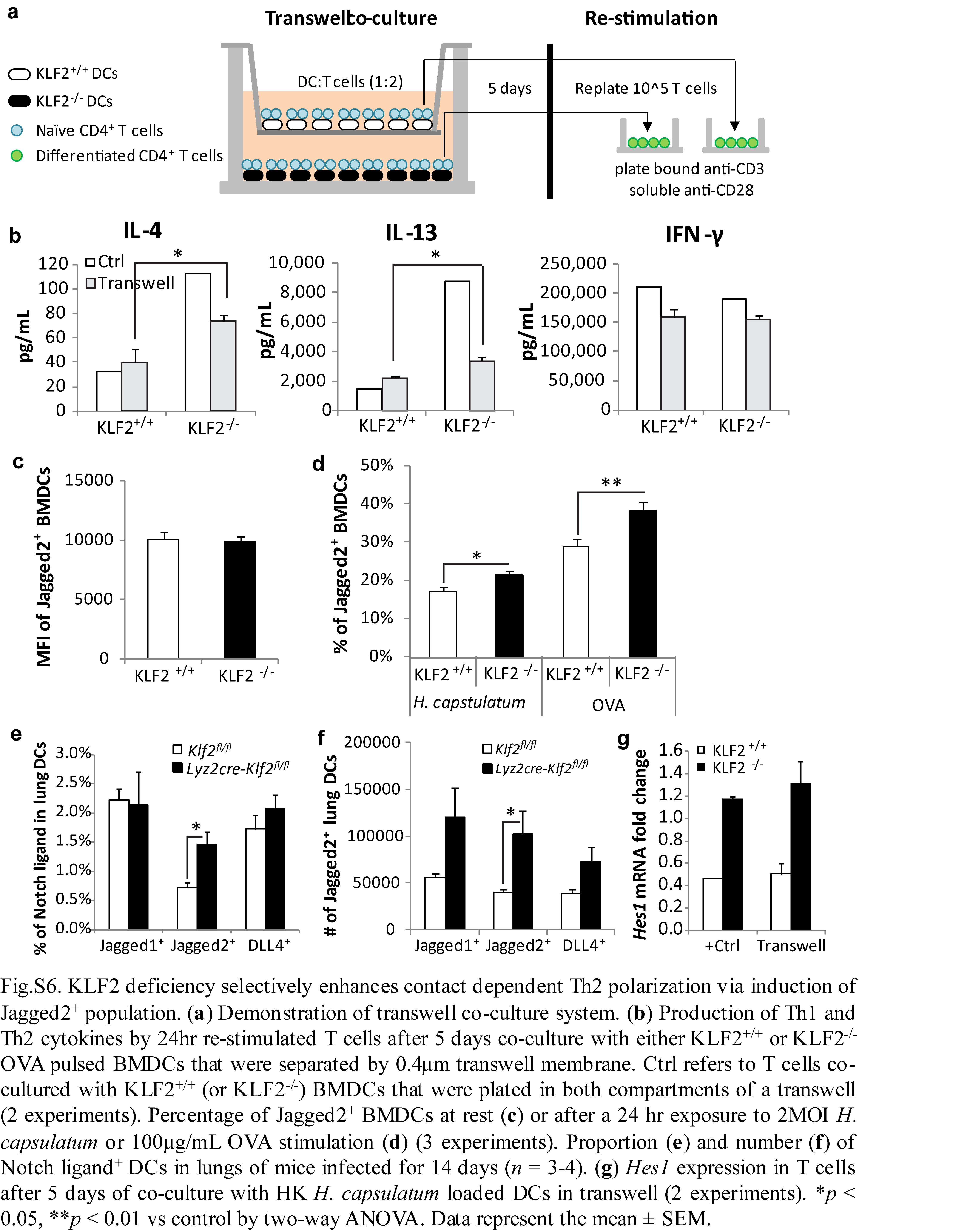

Supplement: Figure S6 — KLF2 deficiency selectively enhances contact-dependent Th2 polarization via induction of Jagged2+ population. (a) Demonstration of transwell coculture system. (b) Production of Th1 and Th2 cytokines by T cells restimulated for 24 h after 5 days of coculture with either KLF2+/+ or KLF2−/− OVA-pulsed BMDCs that were separated by 0.4-µm transwell membrane. Control (Ctrl) T cells were cocultured with KLF2+/+ (or KLF2−/−) BMDCs that were plated in both compartments of transwells (2 experiments). (c and d) Percentages of Jagged2+ BMDCs at rest (c) or after 24 h of exposure to 2 HK H. capsulatum yeast cells/DC or 100-µg/ml OVA (d) (3 experiments). (e and f) Proportions (e) and numbers (f) of Notch ligand+ DCs in the lungs of mice infected for 14 days (n = 3 or 4). (g) Hes1 expression in T cells after 5 days of coculture with HK-H. capsulatum-loaded DCs in transwells (2 experiments). *, P < 0.05, and **, P < 0.01, for comparison with the results for the control by two-way ANOVA. Data represent the mean results ± SEM. Download [file mbo003162840sf6.tif]

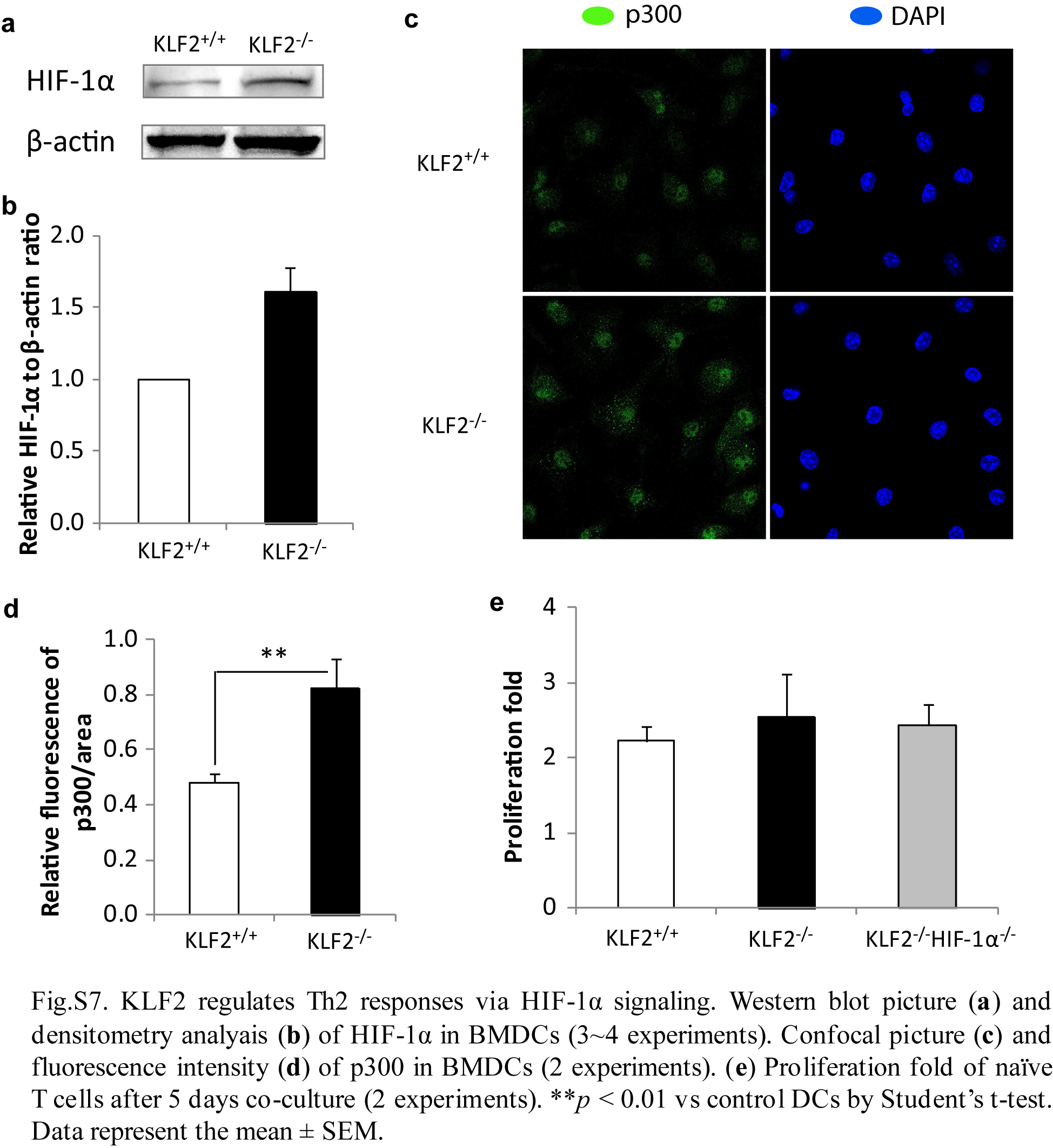

Supplement: Figure S7 — KLF2 regulates Th2 responses via HIF-1α signaling. (a and b) Western blot results (a) and densitometry analysis (b) of HIF-1α in BMDCs (3 to 4 experiments). (c and d) Confocal images (c) and fluorescence intensities (d) of p300 in BMDCs (2 experiments). (e) Fold proliferation of naive T cells after 5 days of coculture (2 experiments). **, P < 0.01 for comparison with the results for control DCs by Student’s t test. Data represent the mean results ± SEM. Download [file mbo003162840sf7.tif]

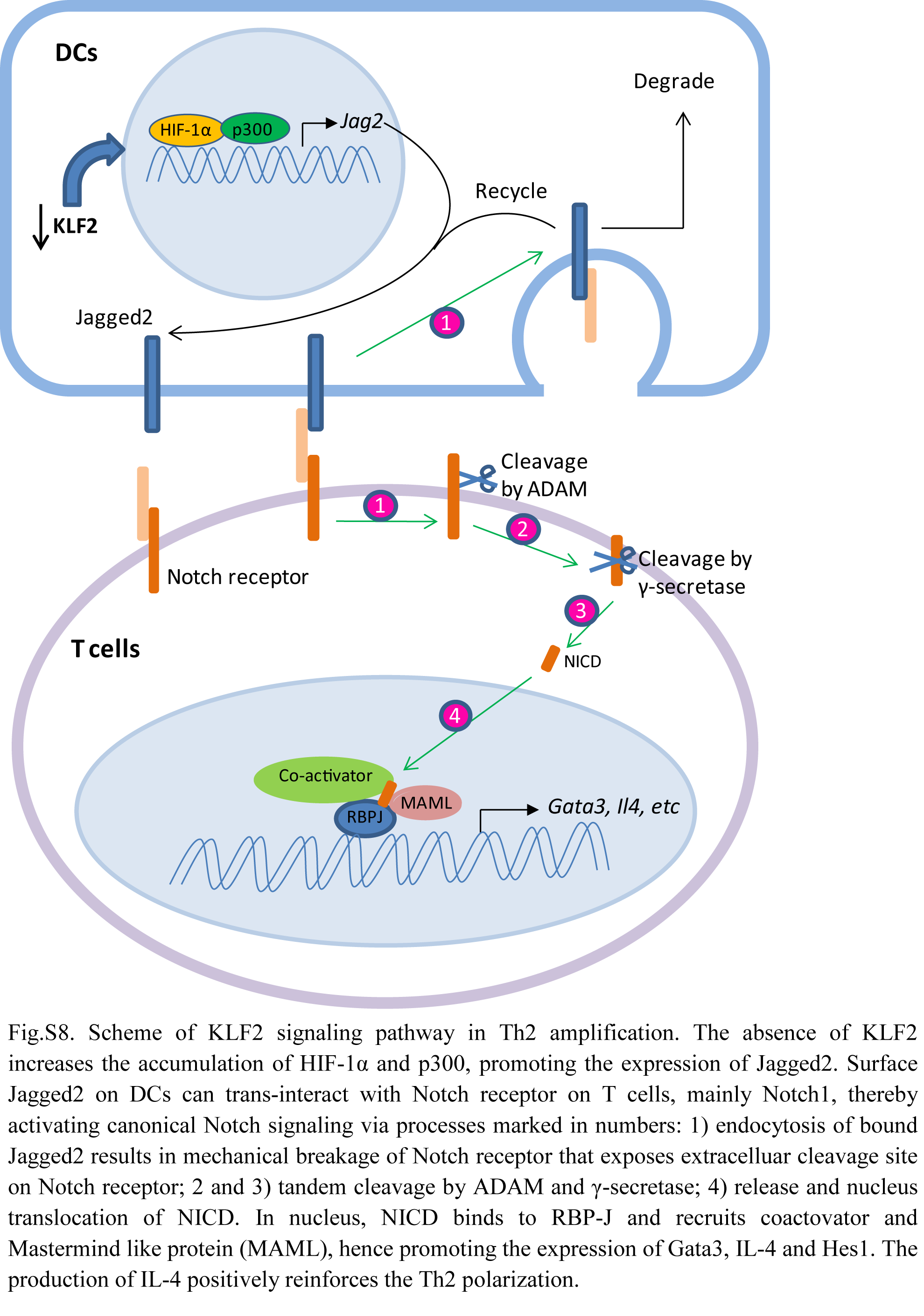

Supplement: Figure S8 — Scheme of KLF2 signaling pathway in Th2 amplification. The absence of KLF2 increases the accumulation of HIF-1α and p300, promoting the expression of Jagged2. Surface Jagged2 on DCs can trans-interact with Notch receptors on T cells, mainly Notch1, thereby activating canonical Notch signaling via processes numbered as follows: (1) endocytosis of bound Jagged2 results in mechanical breakage of Notch receptor that exposes extracellular cleavage site on Notch receptor; (2 and 3) tandem cleavage by ADAM and γ-secretase; (4) release and nuclear translocation of NICD. In the nucleus, NICD binds to RBP-J and recruits coactivator and mastermind-like protein (MAML), hence promoting the expression of Gata3, IL-4, and Hes1. The production of IL-4 positively reinforces the Th2 polarization. Download [file mbo003162840sf8.tif]
